# Promoting versatile vaccine development for emerging pandemics

**DOI:** 10.1038/s41541-021-00290-y

**Published:** 2021-02-11

**Authors:** Joshua T. Monrad, Jonas B. Sandbrink, Neil G. Cherian

**Affiliations:** 1 Faculty of Public Health and Policy, London School of Hygiene and Tropical Medicine, London, UK; 2 Department of Health Policy, London School of Economics, London, UK; 3 Future of Humanity Institute, University of Oxford, Oxford, UK; 4 Medical Sciences Division, University of Oxford, Oxford, UK; 5 The Coalition of Epidemic Preparedness Innovations, Oslo, Norway; 6 Johns Hopkins Center for Health Security, Baltimore, USA

**Keywords:** Vaccines, Vaccines, Medical research, Preclinical research

## Abstract

The ongoing COVID-19 pandemic has demonstrated the importance of rapid and versatile development of emergency medical countermeasures such as vaccines. We discuss the role of platform vaccines and prototype pathogen research in modern vaccine development, and outline how previous pathogen-specific funding approaches can be improved to adequately promote vaccine R&D for emerging pandemics. We present a more comprehensive approach to financing vaccine R&D, which maximises biomedical pandemic preparedness by promoting flexible vaccine platforms and translatable research into prototype pathogens. As the numerous platform-based SARS-CoV-2 vaccines show, funders can accelerate pandemic vaccine development by proactively investing in versatile platform technologies. For certain emerging infectious diseases, where vaccine research can translate to other related pathogens with pandemic potential, investment decisions should reflect the full social value of increasing overall preparedness, rather than just the value of bringing a vaccine to market for individual pathogens.

## Introduction

The COVID-19 pandemic has highlighted the need to proactively and rapidly develop medical countermeasures against novel pandemic pathogens. As an emerging pathogen, the work to develop and trial SARS-CoV-2 vaccine candidates only began after the novel strain was sampled and the genome was sequenced in early January. By the time the World Health Organisation declared a global pandemic in March, many projections suggested that regulatory authorisation and distribution of a vaccine could take up to several years. Less than 12 months later, the first patients have been vaccinated with safe and effective vaccines outside of clinical trials, and immunisation efforts are due to commence globally. The fact that the SARS-CoV-2 vaccine development and authorisation process has been the swiftest in history owes to several factors, including a concerted research effort, unprecedented public and private investments, and overwhelmingly high global incidence. But another key factor that enabled vaccine developers to progress so rapidly has been a set of specific vaccinological insights and technologies developed before the novel coronavirus was even identified. In this article, we examine how to promote modern approaches to shortening the timelines between novel pathogen identification, successful vaccine licensing, and population-level coverage. Because biotechnological knowledge obtained from certain work on existing pathogens can be applied for research on future countermeasures against novel pandemic diseases, a pathogen-specific approach to financing research and development (R&D) fails to create the adequate incentives to invest in flexible approaches that maximise pandemic preparedness. We present a more comprehensive approach to biomedical innovation policy that emphasises vaccine platform development and translatable research on prototype pathogens and join others in calling for increased investment in promising technologies against the next pandemic^1–3^.

Two distinct but complementary approaches stand out as promising pathways to accelerating the development of future pandemic vaccines: the advancement of vaccine platforms and research on prototype pathogens^4,5^. While the term “platform technologies” for biomedical innovation is used in a wide range of contexts^6^, we adopt a working definition stating that a vaccine candidate is platform-based if “an underlying, nearly identical mechanism, device, delivery vector, or cell line [is] employed for multiple target vaccines”^7^. This definition encompasses a range of product types, including vaccines based on viral vectors, recombinant protein expression systems, or nucleic acids, and distinguishes novel platform vaccines from pathogen-specific approaches, such as whole-inactivated or live-attenuated viruses, that are the basis of most currently licensed vaccines.

The other approach for shortening development timelines of future pandemic vaccines is to research certain ‘prototype pathogens’ that may inform future vaccine design for similar pathogens^4,5,8,9^. Under the prototype pathogen approach, ‘investigators would conduct countermeasure research for prototype pathogens, understanding that the prototype may not emerge as a threat but assuming that techniques would be applicable to closely related microorganisms’^3–5,8^. Such research can target prototypic members of viral families, genera or species known to pose risks to humans, prioritised according to their expected structural similarity to the next potential pandemic pathogen. It may, for example, involve identifying conserved antigen epitopes, so that insights into vaccine design may be readily translated to tackling novel pathogens. This work may also inform the development of multivalent or universal vaccines efficacious against related viruses through targeting a conserved antigen. Here, we focus especially on viral pathogens due to their potential for causing global catastrophic pandemics and in this paper, we primarily use the terms ‘prototype pathogen’ and ‘emerging pathogens’ in reference to viruses^3,5^. However, the importance of emerging bacterial, parasitic, and fungal pathogens should not be understated—particularly in light of increasing antimicrobial resistance—and the general approach that we present here can potentially be applied to non-viral emerging pathogens.

## The current landscape for vaccine R&D

### Failures in the market for infectious disease vaccines

While for-profit pharmaceutical companies relying on commercial investments conduct a large share of pharmaceutical R&D—especially late-stage clinical trials—a range of well-understood economic factors cause private investment in infectious disease vaccines to often fall far short of what would be the socially optimal level, compared to other biomedical products. Economic theory and evidence suggest that preventive products tend to be less lucrative than curative therapeutics, even for products with the same potential to generate social value^10^, and the powerful positive externalities that vaccines create by preventing community transmission mean that the demand for vaccines is often lower than the socially optimal level^11^. Consequently, with the exception of a few blockbuster products—such as Pfizer’s multivalent pneumococcal vaccine and Merck’s nonavalent human papillomavirus vaccine, both of which have billion-dollar markets—vaccine sales rarely constitute a large share of revenues for leading pharmaceutical producers. This problem is exacerbated in the case of pathogens primarily affecting populations in lower-income countries with less purchasing power, and in the case of sporadic emerging epidemic pathogens, where a sustained demand for the vaccine is not guaranteed and investments are particularly risky from a commercial perspective^12–14^.

### Existing approaches to creating markets for vaccines

In light of these pervasive and well-understood market failures, several actors have financed vaccine R&D for known diseases with epidemic potential^15^. These include governmental agencies, such as the Biomedical Advanced Research and Development Authority and the National Institutes of Health (NIH) in the United States; supranational bodies, such as the European Commission; and philanthropic foundations, such as the Bill & Melinda Gates Foundation and Wellcome Trust. In the wake of the 2014–2015 Ebola outbreak, the Coalition of Epidemic Preparedness Innovations (CEPI) was founded as a public-private partnership to coordinate funding for vaccines against epidemic diseases. Since then, CEPI has supported the development of numerous vaccines for priority pathogens and worked to ensure global equitable access to novel licensed vaccines^16^.

In addition to the ‘push’ funding (i.e., up-front grants for promising projects) for preclinical and early clinical work, there is a need for ‘pull’ mechanisms (i.e., rewards for output, such as prizes or market guarantees) more suitable for late-stage clinical trials and product development, as well as global mechanisms to finance, manage, and maintain investigational product stockpiles for preparedness. Although certain emerging pathogens like Nipah virus and MERS-CoV may not cause outbreaks that are large enough for conventional efficacy trials, such that licensure may be considered on the basis of data from animal studies^17^, most vaccine development programs will involve phase 3 trials that require significant capital investments. One proposal to create the adequate incentives to complete such trials is the ‘Advance Purchase Commitment’ (APC), which works by having a funder precommit to purchasing doses of a vaccine, conditional on it being successfully licensed and produced^11^. This approach has been successfully employed to facilitate the development of pneumococcal vaccines^18,19^, and purchase agreements are also being employed for SARS-CoV-2 candidates, including through the COVAX initiative from Gavi, CEPI, and the WHO^20^.

While the APC is a promising solution for endemic diseases or widespread active pandemics, where high demand for the vaccine is guaranteed, its applicability for emerging epidemic diseases is often limited by the fact that the mechanism ties developer remuneration to the quantity of procured doses. If an epidemic vaccine is employed early, when case number and geographical distribution are still very limited, it may successfully contain an outbreak before it grows too large, thereby quelling its own market because fewer doses are required^21^. Consequently, the standard APC does not strongly incentivise bringing the product to market proactively to preempt widespread pandemics. As Christopher Snyder and colleagues note, “When the best social outcome determines the worst economic outcome for the developer, there is a strong argument for a non-market intervention”^21^.

To optimally incentivise commercial vaccine developers, funding mechanisms for epidemic vaccines should delink revenues from used doses and instead create a reward that matches the social value of developing vaccines, including both health and economic benefits. Christopher Snyder, Kendall Hoyt, and colleagues from CEPI recently proposed a mechanism with this feature^13^. Focusing on the case of SARS-CoV-2 vaccines, they present a variation of the APC in which the maximum amount paid for successful development is derived from the total social value of deploying a vaccine against SARS-CoV-2, accounting for both the value of saving lives and the economic benefits of mitigating the pandemic. The idea of bringing private incentives better in line with expected social value has been proposed and applied in other contexts^22^, and should be integrated into financing mechanisms for epidemic vaccine R&D beyond SARS-CoV-2^13,21^.

## Market failures arising from spillovers in vaccine research

Combinations of push-funding and pull-funding offer promising solutions to financing both early and late-stage R&D for epidemic vaccines. However, a distinct challenge arises in the context of funding for vaccine platforms and prototype pathogen research, as the total social value of advancing these technologies—including translatable biotechnological insights that increase pandemic preparedness—exceeds the direct value of having a medical countermeasure for any one particular pathogen. In a nutshell, the knowledge obtained from advancing vaccine platforms and applying them to prototype pathogens creates positive technological spillovers that render pathogen-specific financing approaches inadequate. (While the term ‘spillover’ often is used in reference to zoonotic spillovers, we use it here in the manner used in the economics literature, as a reference to the positive externalities of technological development.)

### Positive technological spillovers of platform advancement

Advancing vaccine platforms generates technology that enables rapid pandemic vaccine development. Platform vaccine approaches, such as RNA, DNA, and virally-vectored vaccines, can feature properties such as fast genetic sequence-based design, testing, and rapid and scalable manufacturing. While these new platforms do not yet have the established track-record of classical inactivation or attenuation approaches—and in the case of DNA vaccines, have only been licensed for veterinary use to date despite decades of research^23^—a growing body of preclinical and clinical evidence supports their significant potential.

The viral vector approach has shown promise as the basis of two approved Ebola vaccines—the recombinant vesicular stomatitis virus vaccine from Merck and the human adenovirus 26 and modified vaccinia virus Ankara vaccine from Johnson & Johnson—as well as multiple vaccine candidates for SARS-CoV-2, including the chimpanzee adenovirus vector vaccine from Oxford-AstraZeneca. Similarly, several SARS-CoV-2 vaccine candidates are based on nucleic acid platforms, including those from NIH-Moderna and BioNTech-Pfizer.

Because vaccines based on the same platform may only differ in the sequence encoding the target antigen, preclinical, and early-stage clinical work on one pathogen can inform the general optimisation, formulation, and delivery of the platform, meaning that platform-based vaccine candidates have the potential to reach the clinical stage of testing faster than more traditional vaccine candidates^4,9,24^. Furthermore, the first phase 3 clinical data on the efficacy of these novel nucleic vaccine platforms does not only indicate their suitability to tackle the pathogen in question but also for a range of pathogens with similar correlates of protection. Additionally, manufacturing facilities constructed for the production of a specific nucleic acid-based vaccine may be repurposed for related vaccines, and experience with large scale manufacturing will directly translate to further optimisation of speed and cost of production of future vaccines based on the same platform. The ongoing development of SARS-CoV-2 vaccines provides evidence for the idea that investing in platforms is a way to enable a more rapid response. The NIH-Moderna, BioNTech-Pfizer, and Oxford-AstraZeneca vaccines for which there is efficacy data from phase 3 trials as of December 2020—a record speed by historical standards—are all based on platform approaches. Moreover, the Oxford-AstraZeneca vaccine, as well as the vaccines from Inovio and Imperial College London that are currently in clinical trials, are based on platforms that CEPI has funded over the past few years^16,25^.

While it remains to be seen whether any of the vaccines against SARS-CoV-2 will provide long-term protection, the fact that platform technologies are behind some of the first candidates to prove effective against the novel coronavirus suggests that efforts to advance these approaches over the past years have yielded significant social value. Crucially, given the various advantages and disadvantages of different platforms, and the potential variation in their usefulness against pathogens with different tissue tropism and susceptibility to humoral or cellular immune responses, it will remain critical to advance a broad portfolio of platforms. For instance, RNA and viral vector-based vaccines can induce robust cellular immune responses that may be necessary for effective immunity against enveloped viruses with complex pathogenesis such as poxviruses or filoviruses, while virus-like particles might be most suitable to induce neutralising antibody responses against non-enveloped viruses^5^. Additionally, potential antigen-specific concerns such as disease enhancement or induction of autoimmunity must be addressed for each new vaccine, largely independently from previous experience with other vaccines based on the same platform, while concurrently generating clinical (human) data supporting the versatility of their underlying platforms.

### Positive spillovers of prototype pathogen vaccine development

Applying vaccine platforms to prototype pathogens within each of the main families of viruses known to cause human infection may be critical for enabling fast development of vaccines in the face of an emerging pandemic^5^. Work on one pathogen may speed up the development of a vaccine against a novel pathogen, as was the case when prior work on yellow fever, Japanese encephalitis virus, dengue, and other members of the *Flaviviridae* family accelerated the emergency development of vaccine candidates for Zika virus^4,5,26^.

The case of earlier research on human β-coronaviruses informing SARS-CoV-2 vaccine development provides an instructive example of how such technological spillover effects may manifest. While the spike protein (S) would have likely been a clear choice of antigen based on its similarity to the cell entry glycoproteins of other viruses, work on coronaviruses corroborated that this major surface protein is indeed a prime target for induction of neutralising antibodies^27^.

Previous work on respiratory syncytial virus (RSV), parainfluenza virus, and MERS-CoV has shown that prefusion-stabilised immunogens may improve neutralising antibody responses against enveloped viruses^28–30^. Before the pandemic, researchers at the U.S. NIH identified two proline (2P) substitutions at the apex of the central helix and heptad repeat 1, which stabilise the spike protein of MERS-CoV, SARS-CoV, and HCoV-HKU1 in its prefusion conformation^29,31^. This prefusion stabilisation has been applied to the SARS-CoV-2 spike protein, and this S-2P prefusion-stabilised spike protein is used in a range of vaccines candidates, including those by NIH-Moderna, BioNTech-Pfizer, Novavax, and Johnson & Johnson, which exemplifies how previous research on antigen optimisation, including for MERS-CoV, has been important for SARS-CoV-2 vaccine development^32–35^. Additionally, a similar lipid nanoparticle formulation to that of the mRNA-1273 vaccine was tested for a MERS-CoV S-2P mRNA vaccine in mice^27^, demonstrating that the S-2P encoding mRNA was able to induce protective immunity against MERS-CoV and indicating that similar approaches might work for SARS-CoV-2 infection. Furthermore, the mouse model of SARS-CoV-2 used in this study, which is based on CRISPR/Cas9-induced mutations in the dipeptidyl peptidase 4 receptor, was initially developed for MERS-CoV^27,36^. Indeed, the generation of animal models is critical for enabling preclinical product development and should be an important part of prototype pathogen work. Early efficacy data show that the rapidly developed SARS-CoV-2 vaccines based on the pre-fusion-stabilised spike protein are efficacious, which suggests that technical spillovers from preliminary MERS-CoV work have successfully accelerated SARS-CoV-2 vaccine development.

Clinical work on a MERS-CoV vaccine has also sped up the clinical development of a SARS-CoV-2 vaccine; safety data from a phase 1 human trial with a virally vectored chimpanzee adenovirus platform (ChAdOx1)-based vaccine encoding the MERS-CoV spike glycoprotein enabled fast commencement of large-scale clinical trials of the ChAdOx1-based AZD1222 vaccine, which encodes the SARS-CoV-2 spike glycoprotein^37,38^. Similarly, prior work on a DNA plasmid vaccine for MERS-CoV aided the development of the SARS-CoV-2 vaccine that Inovio Pharmaceuticals as of December 2020 has moved into a phase 2 trial^39^. Clinical efficacy trials of vaccines against prototype pathogens may be critical for identifying the correlate of protection for different viral families, i.e., what extent of induction of neutralising antibody responses versus cellular immunity is needed by a vaccine to be protective. This may help to inform platform choice, antigen selection, and vaccine formulation and delivery when starting vaccine development on a novel-related pathogen.

These examples of SARS-CoV, MERS-CoV, and HCoV-HKU1 research being translated to the context of SARS-CoV-2 highlight how work on prototype pathogens creates positive scientific and technological spillovers. Some of these spillovers, such as the promising nature of the spike glycoprotein as a target antigen or information on animal models of infection, are likely to be published widely and can therefore benefit research efforts for other vaccine-preventable diseases. In this case, the generated knowledge is a clear example of a positive spillover that creates a need for public intervention into the market for research and development. However, this relies on the results of translatable work on prototype pathogens—such as insights into antigen optimisation—being accessible to public use. Therefore, public funding of prototype pathogen work should seek to promote research that generates openly accessible and translatable insights as far as practicable, while also judiciously taking advantage of generating proprietary intellectual property. Even in the cases where a proprietary insight might primarily benefit the originating organisation, such as early preclinical evidence and safety data from clinical trials, the research remains worthy of subsidy because society benefits from having developers that are better prepared to respond to emerging infectious diseases.

Beyond novel platforms, the prototype pathogen approach may also be applied to live attenuated viral and inactivated virus vaccines, where insights on antigen design, attenuation, and optimisation, as well as purification and manufacturing process steps, are potentially transferable to development of vaccines for related pathogens. Therefore, even for more classical vaccines, research, and development investments may create knowledge spillovers that are not pathogen-specific. For instance, a novel synonymous codon replacement attenuation approach has been applied to both influenza A virus and RSV^40^.

### The missed opportunity of pathogen-specific approaches to financing

Due to the technological spillovers from platform advancement and prototype pathogen research, pathogen-specific approaches to funding lack incentives that maximise biomedical preparedness for novel emerging pathogens. For preclinical and early clinical stages, grant-based funding can offset commercial risk for vaccine developers by covering the costs of research and development up to a certain point. But given the high opportunity costs of diverting resources from more lucrative domains of pharmaceutical development, the prospect of merely covering costs may not induce strong enough interest from industry^41^. And because even preclinical and early-stage clinical work on vaccine platforms and prototype pathogens can yield valuable technological know-how, financial incentives must be structured such that companies can expect a competitive return on investment by reaching certain milestones along the R&D pathway, even if an investment does not result in a product licensed by regulators.

Pull-mechanisms can solve the problem of incentivising late-stage vaccine development for epidemic diseases, if their prize amount is derived from the expected harm caused by known pathogens. While uncertainty exists around whether certain pathogens are even likely to cause widespread epidemics, epidemiological, and economic modelling can be employed to estimate, at least to a first approximation, the optimal risk-adjusted rewards for successful development of vaccines against known emerging infectious diseases. However, by focusing on the value of addressing individual pathogens, such solutions will undervalue the advancement of platform-based vaccines as well as their application to certain prototype pathogens.

Consider, for example, a funder looking to support work on one of the WHO’s priority pathogens^42^. If their funding decisions were conceived exclusively to address that pathogen, they would, all else equal, offer the very same financial incentives for pathogen-specific approaches (such as live-attenuated or whole-inactivated vaccines), as they would have for platform-based vaccines. Such a funding decision would be made in spite of the fact that the latter kind of project could create substantial positive spillovers by advancing the underlying platform technology. While firms or other entities do of course benefit from advancing their platform technologies, this benefit can only be realised insofar as successful product development for other pathogens is sufficiently commercially lucrative in the first place; a condition that is unlikely to be met given the numerous failures in the market for vaccines outlined above. Consequently, as the number of potential applications of an innovative platform increases, so does the magnitude of the gap between private value and social value. In other words, the more useful a platform technology for the development of vaccines against a wide range of viruses—for instance through induction of both neutralising antibodies as well as a robust cellular immune response—the more complementarities there are between research on one pathogen and research on other emerging pathogens, and the stronger is the market failure and the justification for intervention in the form of non-market incentives.

The case of MERS-CoV provides an illustrative example of how the same problem exists in the case of prototype pathogens. Suppose that a funder in 2019 had contemplated supporting the development of a vaccine for MERS-CoV; a virus that had already been identified as a target for prototype research on the *Coronaviridae* family by researchers at the U.S. NIH^5,8^. If the funder did not consider the generalisable insights that may arise from work on MERS-CoV, they may underinvest in such research. To be clear, the high fatality rate of MERS and the history of significant nosocomial outbreaks certainly warranted support for vaccine development, and the virus was rightly identified as a priority pathogen by both the WHO and CEPI^42^. However, at the time, certain epidemiological features of the virus could have appeared to weigh against larger investments: less than three thousand cases were reported between 2012 and 2019—the majority of which were in just one country, Saudi Arabia—and human-to-human transmission appeared limited to close-contact healthcare settings^43^. Given these transmission characteristics, any estimates of the value of a MERS-CoV vaccine may have had their upper bounds defined by the apparent lack of truly pandemic potential. With the benefit of hindsight, however, we know that prior work on platform-based vaccines for MERS-CoV has offered highly valuable insights for SARS-CoV-2. Thus, any financing scheme that did not account for this complementarity would have undervalued R&D for MERS-CoV platform vaccines.

Going forward, it will be critical to identify the prototype pathogens for which proactive research will yield the greatest benefits for biomedical pandemic preparedness. For example, researchers from the U.S. National Institute of Allergy and Infectious Diseases have highlighted Nipah virus (NiV) as a prototype pathogen for the *Henipavirus* genus in the *Paramyxoviridae* family, and CEPI has invested in early vaccine development programs for NiV^5^. The regular emergence of NiV outbreaks, involving some human-to-human transmission, and relatively high mutational rate of RNA viruses raises the possibility that we will see a future pandemic from a virus related to the current NiV that has acquired greater transmissibility^44,45^. Since work on vaccines for NiV, such as generalisable structure-based approaches to antigen stabilisation and immunogen development, can apply to other henipaviruses or paramyxoviruses^46^, it is critical that pharmaceutical interest in such work does not hinge exclusively on the commercial value of bringing a single henipavirus vaccine to market.

## Solutions

### Creating stronger commercial incentives

To improve overall pandemic preparedness, pathogen-specific approaches to funding R&D should be complemented by efforts that maximise our collective ability to address novel unknown pathogens. As the example of SARS-CoV-2 shows, especially preclinical and early-stage clinical work can be highly valuable in demonstrating the viability of a platform or improving antigen selection and optimisation for prototype pathogens. The work of CEPI provides a model approach for funding this kind of R&D, following their long term objective “to transform a selection of the platforms funded … into a sustainable toolbox of platform technologies [that] could be ready for response”^47^. To facilitate the advancement of multipurpose platforms, CEPI requires potential grantees to propose plans for generating preclinical safety, immunogenicity and efficacy data for three different pathogens in addition to their plans for performing a Phase I clinical trial for at least one selected antigen^47^. Moreover, CEPI accounts for the complementarity between research on different pathogens by encouraging grantees to develop their platform vaccines against known prototype pathogens. By considering how technological spillovers increase the expected social value of certain research when prioritising between projects to fund, governments and philanthropists can spur early-stage research leading to potentially generalisable insights and allow for a longer-term research agenda into important areas, that may be relatively neglected by the commercial pharmaceutical sector. Importantly, investments in prototype pathogen research and vaccine platform advancement are also complementary, as the value of understanding prototype pathogens is greater if that knowledge can be applied through flexible platforms, and vice versa.

For pull-mechanisms designed to induce successful product development, the prize must account for the indirect benefits of advancing platform vaccines and researching prototype pathogens. One promising approach would be to modify the advance purchase agreement variation recently developed by Snyder and colleagues^13^. Since, their model sets the potential prize paid to vaccine manufacturers as a function of the expected social value of a successfully developed vaccine (the funder’s “demand”), it could be adapted by extending this derivation to include a term for the indirect value of technological spillovers. This would require quantifying the benefits of various technological and scientific advances by considering the relative advantages of various platforms^7^, the pandemic characteristics of different pathogens^3^, and the concrete potential for shortening vaccine development timelines or increasing the likelihood of success. While obtaining an exact quantitative valuation may be complex, it is possible to evaluate the relative merits of various scientific advances and set financial incentives accordingly. For example, reward amounts should be greater for the first time that any manufacturer successfully demonstrates safety and efficacy in phase 3 trials for any given platform or prototype pathogen, since such data may inform further optimisation of the platform or shed light on correlates of protection for related viruses.

In considering the relative merits of different vaccine platforms, funders should also consider potential dual-use concerns associated with some kinds of biomedical research. For example, certain research on viral vectors, particularly work which involves viral engineering to evade antivector immunity, may lead to insights associated with dual-use risk. Such risks are a negative externality borne by all of society rather than just the agent undertaking the research. By explicitly incorporating this risk into funding decisions—e.g., by announcing that larger financial rewards are awarded to robust approaches—funders can incentivise the safe and secure development of novel synthetic biological methods. Recent work by Sebastian Farquhar and colleagues shows how quantitative estimates of dual-use risks can be obtained through either a market-based insurance model or a centrally commissioned risk assessment^48^.

### Alternative approaches to accelerating R&D

Beyond creating market-oriented financial incentives, governments and philanthropists can accelerate biomedical R&D by supporting and coordinating precompetitive collaborations between industry stakeholders. By pooling technical expertise and collaborating towards shared advances for vaccine platforms and prototype pathogens, such as improved assays, animal models, and manufacturing processes, developers can benefit from the downstream R&D outputs that apply to a wide range of vaccine products, including both novel platforms and classical vaccine approaches. This approach has been applied successfully in the form of the Biomarkers Consortium, the Patient-Reported Outcomes Consortium, and the Innovative Medicines Initiative, and key proponents of the prototype pathogen approach have called for the formation of scientific consortia for work on universal vaccines and novel vaccine-delivery approaches^5^. The World Health Organisation is well-positioned to play a normative role in facilitating consortia efforts and knowledge sharing, as are civil society organisations as crucial advocates for equitable access. Moreover, public sequence repositories such as the Global Initiative on Sharing Avian Influenza Data, where early SARS-CoV-2 sequences were shared, can facilitate faster commencement of global vaccine development efforts.

Given the profound benefits of developing novel vaccine technologies, governments may also be in an ideal position to take a more proactive approach. Publicly financed research institutions, including universities, already contribute considerably to vaccine development by generating non-proprietary insights into fundamental immunology and vaccinology. By prioritising platform vaccines and prototype pathogen research, universities and public agencies can play a critical role in performing early fundamental research and advancing adaptable vaccine technologies. Moreover, in addition to undertaking crucial basic scientific research, public, and non-profit research institutions can lead the way through all stages of vaccine development. Examples include R&D efforts spearheaded by the U.S. government and military, which led to the successful development of numerous vaccines in the 20th century^49^. More recently, the Public Health Agency of Canada designed the rVSV Ebola vaccine that was since licensed to the biotechnology firm NewLink Genetics and subsequently acquired by Merck before it was authorised for use by regulators in the EU, North America, and Africa. Moreover, the public Instituto Butantan in Brazil has played a central role in the late-stage development of affordable vaccines for dengue and influenza^21,50^. Similarly, public agencies have been central in the development of various SARS-CoV-2 vaccines, including the U.S. NIH and the Chinese Wuhan Institute of Biological Products^25,51,52^. In addition to prioritising the most socially valuable R&D targets, public involvement in vaccine development can promote equitable and affordable vaccine access through volume and pricing commitments for developing countries.

## Conclusion

Biomedical pandemic preparedness requires prudent investments in R&D for vaccine platforms and prototype pathogens, which account for the knowledge gains that aid vaccine development for multiple pathogens, including novel viruses with pandemic potential. Governments should take an active role in leading development efforts with positive technological spillovers, through publicly financed research, product development partnerships, and facilitated precompetitive partnerships. While we have primarily focused on viruses and novel vaccine technologies here, this overarching argument is applicable to other emerging pathogens, including drug-resistant bacteria, as well as more classical vaccine approaches. Indeed, given the extensive prior experience with established safety and lasting protection profiles from large scale immunisation campaigns—such as for measles, yellow fever virus, polio, and influenza—these vaccines have some valuable advantages relative to novel platform approaches and may be an important component in the biomedical preparedness arsenal. Additionally, at least in the short term, manufacturing and distribution capacity may be greater for more established vaccine types, increasing their relative importance for the ongoing immunisation efforts against SARS-CoV-2. Crucially, efforts to accelerate R&D must be complemented by other policies that can shorten the timeline between pathogen identification and population coverage, such as regulatory innovation to facilitate platform-based vaccine licensure, development of multivalent or universal vaccines, and efforts to ensure global, equitable vaccine access^53^. Additionally, to prepare for scenarios where conventional clinical trials may be unethical or infeasible, there is a need for regulatory innovation to facilitate potential emergency use authorisation on the basis of efficacy data from animal models and in some cases controlled human infection models^17,54,55^. Finally, realising the full potential of platform vaccines requires overcoming challenges in manufacturing and delivery, such as improving nucleic acid vaccine thermostability or creating cold chain storage solutions and developing devices for DNA vaccine electroporation. As the enormous human and economic costs of the COVID-19 pandemic make painfully clear, investments that enable more rapid development of medical countermeasures can provide potential net economic benefits on the order of trillions of dollars^13^, paying for themselves many times over and enhancing global health security in the process.
